# Artificial intelligence and small-business competitiveness in an emerging economy

**DOI:** 10.3389/frai.2026.1927049

**Published:** 2026-10-09

**Authors:** Layli Navruz-zoda, Nurulla Fayzullaev, Kakhramon Madrakhimov, Aajaz Ahmad Hajam, Razzaqbergan Jumaniyozov, Dilora Saydamenova, Inomjon Yusubov

**Affiliations:** 1 Department of Marketing and Management, Bukhara State University, Bukhara, Uzbekistan; 2 Department of Economics Urganch RANCh University of Technology, Khorezm, Uzbekistan; 3 Department of Business Administration, Mamun University, Khiva, Uzbekistan; 4 International Foundation Programme, Tashkent International University of Education, Tashkent, Uzbekistan; 5 Department of Business Administration, Mamun University, Tashkent, Uzbekistan; 6 Diplomat University, Tashkent, Uzbekistan; 7 Kimyo International University, Tashkent, Uzbekistan; 8 Department of Economics, Urgench State University named after Abu Rayhan Biruni, Khorezm, Uzbekistan

**Keywords:** AI adoption, ARIMA forecasting, artificial intelligence, digital transformation, emerging economy, small business, SME competitiveness, Uzbekistan

## Abstract

Small and medium enterprises (SMEs) are the driving force of developing economies, yet they remain limited in resources to implement artificial intelligence (AI), and there is little empirical evidence that AI adoption is associated with small business growth in the region, particularly in Central Asia. This research makes two contributions. First, we propose a concise framework that links classes of AI technologies to SME competitiveness features and identifies machine learning, natural language processing, and generative AI as low-cost, quick-return priority areas for resource-constrained firms. Second, using 16 years of data (2010–2025) for the Bukhara region of Uzbekistan, we estimate ARIMA models for each indicator and forecast eight small business indicators to 2030. Small business output is projected to grow at a steady rate of approximately 16% by 2030, with digital adoption and preferential credit support emerging as indicators associated with long-run growth. Forecast accuracy is high for the human-capital indicator and acceptable for output. The evidence is associational rather than causal: the framework and the forecasts converge on a single, cautiously formulated proposition—the adoption of low-cost AI is measurably associated with the sustainable competitiveness of SMEs, providing a practical, evidence-based starting point for regional AI policy in developing countries.

## 1 Introduction

Small and medium-sized enterprises (SMEs) are the backbone of developing economies, providing a large share of jobs and a large share of output, but they remain structurally constrained by inadequate financing, staff shortages, and fierce competition from large players (OECD, 2025; Shahadat et al., 2023). In Uzbekistan, small businesses accounted for about 54% of GDP and nearly three-quarters of employment in 2024, making the competitiveness of this sector a national development priority (UNDP, 2025; Yakhshiboev, 2025). Classical competitive theory explains where a firm’s competitive advantage comes through cost or differentiation position and the five forces dynamics (Porter, 1980), valuable and scarce resources (Barney, 2000), and dynamic capabilities that reconfigure these resources despite change (Teece et al., 1997; Chesbrough, 2003) - but it does not specify the role of artificial intelligence (AI) and its predecessors.

Artificial intelligence is increasingly seen as a direct solution to these limitations, automating repetitive processes, personalizing customer interactions, and supporting data-driven decision-making with fewer resources (Cimino et al., 2025; Bastida et al., 2025). Recent reviews point to AI as a strategic and operational enabler of innovation and competitiveness in SMEs (Yesuf and Fields, 2025), and empirical studies link the use of AI in marketing to improved performance of SMEs in developing countries (Abrokwah-Larbi and Awuku-Larbi, 2024). The rapid spread of generative AI has accelerated this transformation across all business functions, from content creation to talent acquisition, analytics, and sustainable management practices (Su and Yang, 2023; Garcia and Kwok, 2025; Do et al., 2025; Li and Cheng, 2025; Nishanthi et al., 2025; Espegren and Hugosson, 2025; Ahmad et al., 2025).

However, the adoption of digital technologies is uneven and the factors shaping it are controversial. Technology-organization-environment studies identify digital infrastructure, organizational readiness, leadership, competitive pressure, and regulation as determinants (Faiz et al., 2024; Badghish and Soomro, 2024; Hoang, 2024; Shao et al., 2025), while digital transformation studies find that leadership and dynamic capabilities mediate increased competitiveness (Gyamerah et al., 2025; Egala et al., 2024; Luo et al., 2023). However, the literature has disproportionately focused on developed economies, leaving SMEs in developing economies, where constraints and opportunities differ, relatively understudied (Yesuf and Fields, 2025; OECD, 2025).

Moreover, much of this evidence is cross-sectional, firm-level, and concentrated on governance and sustainability outcomes rather than on regional growth. Recent studies link firm-level AI capability to eco-innovation in Asian firms, with national digital-economy development strengthening the effect (Mansour et al., 2026a); to board-level governance and corporate tax behavior (Mansour et al., 2026b); to green-innovation efficiency under ESG governance in emerging economies (Mansour et al., 2026c); and to risk reduction through AI disclosure (Saleh et al., 2026). Valuable as they are, these studies stop at the firm boundary and rarely connect adoption to regional outcomes.

Thus, two questions remain open. First, which AI technologies generate the best returns for resource-constrained firms and through which channels can competitiveness be improved? Second, is the adoption of AI/digital technologies empirically linked to small business growth at the regional level, or is this largely a rhetorical question? Recent work highlights the importance of case studies from emerging economies—digital monitoring of national programs in Kazakhstan (Uandykova et al., 2025), generative AI for hiring in Indonesia (Ramaditya et al., 2026), insightful monitoring of smart cities in Kazakhstan (Zhukabayeva et al., 2026), and machine learning-based infrastructure planning (Anand and Grama, 2026), and AI-based decision support in the public sector (Fischer-Abaigar et al., 2024; Myagmar-Ochir and Kim, 2023). However, these studies have largely focused on individual applications rather than linking the structure of AI implementation to measurable regional outcomes. To our knowledge, no study has coupled an operational AI-technology framework with regional time-series evidence for Central Asia; that framework-to-evidence link is the specific gap this paper fills.

Quantitative evidence of this relationship can be obtained through transparent time-series forecasting. Autoregressive integrated moving average (ARIMA) models remain a standard, interpretable framework for short-term economic forecasting (Kontopoulou et al., 2023) and have been applied to analyze regional growth in emerging economies (Balungu and Kumar, 2024). Uzbekistan provides a prime example: its National AI Strategy for 2024 envisages allocating approximately US$1.5 billion annually for AI products and services by 2030, although the use of these funds at the SME level is limited by the availability of funding, skills, and information (UNDP, 2025; Yakhshiboev, 2025).

This research article examines both issues for the emerging economy of Bukhara, Uzbekistan. We (i) propose a concise framework that maps classes of AI technologies to SME competitiveness functions and ranks them by time and cost value, and (ii) provide quantitative evidence using ARIMA models to forecast eight regional SME indicators, including digital adoption, to 2030 and identify which indicators act as long-term determinants of outcomes. The two components are deliberately integrated: the framework generates a testable proposition (affordable AI adoption is associated with competitiveness); the digital-adoption index (X7) operationalizes that proposition; and the ARIMA analysis provides an associational—not causal—test of it against regional data. Rather than developing a new AI algorithm, our contribution is this framework-to-evidence link. Section 3.1 delivers contribution (i); Sections 3.2–3.4 deliver contribution (ii).

## 2 Methods

This study combines a synthesis based on qualitative literature with transparent univariate time series analysis. No new AI algorithms have been developed; the forecasting tool is a classic ARIMA.

### 2.1 Study design

A regional retrospective and prospective study of the small business (SME) sector consists of two parts. The first is a conceptual comparison of artificial intelligence (AI) technologies with SME competitiveness functions, synthesized based on the literature on applied AI activities. The second is an empirical part, modeling eight regional indicators for the period 2010–2025 and projecting them to 2030. Both parts are linked by a single indicator—digital technology adoption (X7), which serves as a general indicator of AI/digital technology adoption.

### 2.2 Data and variables

The empirical analysis uses annual observations for the small business sector in Bukhara for the period 2010–2025 (*n* = 16 years), compiled from official statistics of the Republic of Uzbekistan (national and regional statistical reports). One dependent variable and seven covariates are collected. The dependent indicator is aggregate small-business output (Y, UZS bn); the seven covariates are investment in fixed capital (X1), production volume (X2), gross value added (X3), and innovation-related expenditure (X4), all in UZS bn; the number of small-business employees with higher education (X5, persons); the volume of preferential bank credits to small businesses (X6, UZS bn); and a digital-technology-adoption indicator (X7), the last serving as an aggregate proxy for firm-level AI/digital uptake. All monetary series are expressed in current Uzbek soums (billions of Uzbek soums). We note three caveats to the data that affect interpretation: (i) the 2025 data are partly estimated/initial baseline values; (ii) there are significant structural breaks in some series between 2023 and 2025 (e.g., the X1 investment series drops sharply in 2023 and then rises sharply in 2024); and (iii) the indicators represent aggregated sector data rather than individual firm-level indicators, so X7 is a proxy rather than a direct measure of AI adoption by individual firms. X7 is drawn from official regional statistical reporting on the innovation and digital-technology activity of small businesses in Bukhara province and is expressed in per cent (2010 = 61; 2025 = 145). A published component-level methodology (constituent indicators and weights) is not available for this series; X7 is therefore treated as a broad official measure of digital/innovation activity rather than a validated composite index. It captures digital adoption broadly and cannot isolate AI-specific uptake or distinguish between technology classes; both constraints are acknowledged in the Limitations. Throughout the paper, “SME” denotes the general enterprise category, while “small business” denotes the officially defined small-business sector of Uzbek regional statistics, which is the unit of measurement here.

### 2.3 Conceptual AI-for-SME framework

The framework is explicitly hypothesis-generating. It was developed through a structured narrative review of the applied AI-in-SME literature (2019–2025; Scopus- and Web of Science-indexed studies and authoritative policy reports covering machine learning, deep learning, natural language processing (NLP), generative AI, and related methods in small firms), with sources included when they reported the business function, implementation cost, or time-to-value of a specific AI technology class. Each of the nine technology classes is aligned with a key SME function across six operational areas (marketing and sales, customer service, finance, logistics and supply chain, manufacturing, and HR) and assessed based on criteria such as quality, cost, and time value. These assessments are expert/literature-based, not quantitative; therefore, the resulting “entry level” (machine learning, NLP, generative AI) is not an empirically based assessment, but rather a reasonable prioritization. Tier assignments follow the cost and time-to-value evidence reported in this literature: the affordability and rapid returns of ML, NLP, and generative AI for SMEs (OECD, 2025; Yesuf and Fields, 2025; Abrokwah-Larbi and Awuku-Larbi, 2024; Garcia and Kwok, 2025), versus the infrastructure and skill requirements of computer vision, deep learning, and autonomous systems (Zhukabayeva et al., 2026; Anand and Grama, 2026). The framework yields three testable propositions: (H1) entry-tier technologies show the highest adoption among resource-constrained SMEs; (H2) entry-tier adoption is associated with faster competitiveness gains than advanced-tier adoption; and (H3) at the regional level, aggregate digital adoption co-moves with small-business output. The empirical analysis below can address only H3; H1-H2 require firm-level data and are stated for future research. This component forms a hypothesis: it supports the proposition that the use of low-cost AI promotes competitiveness, but does not by itself test this hypothesis.

### 2.4 Time-series forecasting model

Visual and descriptive analysis shows that the regional series are not stationary, with persistent upward trends and a structural break between 2023–2025. Therefore, we used the Box-Jenkins autoregressive integral moving average (ARIMA (*p*, *d*, *q*) model, where the integration order of *d* differences forces the series to (approximate) stationarity before constructing the ARMA (*p*, *q*) structure. Each indicator is modeled independently (separate univariate model for each series); no cross-correlations or multivariate structures are used.

Let Ω_*T* = {*x*_*t*, *u*_*t*: *t* ≤ *T*} denote the information available up to period *T* for a series *x*_*t* with shocks *u*_*t*. The *τ*-step-ahead forecast *x̂*_{*T* + *τ*}_ is taken to be optimal in the mean-squared-error sense, as given by Equation 1:


$$\min\;E[{\left(x_{\left\{T+\tau \right\}}-{\overset{⌢}{x}}_{\left\{T+\tau \right\}}\right)}^{2}]$$ (1)

The forecast is constructed recursively: future shocks are replaced by their zero expectation, and earlier forecasts substitute for unknown future values, sequentially for *τ* = 1, 2, …. For a stationary process, this implies long-horizon convergence to the unconditional mean, as stated in Equation 2:


$$\lim_{\left\{\tau \to \infty \right\}}{\overset{⌢}{x}}_{\left\{T+\tau \right\}}=E[x\_t]$$ (2)

so that ARIMA forecasts are informative mainly at short horizons. A five-year horizon (2026–2030) is consistent with this short-horizon property. For each indicator, the orders (*p*, *d*, *q*) were selected automatically by minimizing the Akaike Information Criterion (AIC) over candidate specifications. The short sample (*n* = 16) is addressed by design rather than ignored: candidate orders are restricted to parsimonious low-order specifications (*p*, *q* ≤ 2); the horizon is capped at 5 years, where ARIMA is most informative; and full residual diagnostics are reported for every series. With so few annual observations, flexible machine-learning forecasters risk overfitting, which is why a transparent ARIMA baseline is the appropriate choice for short regional series (Kontopoulou et al., 2023; Balungu and Kumar, 2024).

### 2.5 Forecast evaluation and residual diagnostics

Each fitted model was assessed using two metrics. Model goodness of fit and parsimony were summarized using the AIC and Bayesian Information Criterion (BIC). In-sample forecast error was summarized using the mean absolute error (MAE), root mean square error (RMSE), and mean absolute percentage error (MAPE); MAPE is presented as a basic, scale-free measure of accuracy. The adequacy of the residuals was tested using the Ljung-Box test for residual autocorrelation (high *p*-values indicate white noise residuals) and the Jarque-Bera (JB) test for residual normality (low *p*-values indicate deviations from normality, typically caused by outliers or abnormal values). These diagnostics are provided for each metric to help readers assess which forecasts are reliable and which (e.g., an investment series with a high MAPE) require caution.

The claims that specific covariates act as “determinants” of the outcome are based on the descriptive interaction of each covariate with *Y* over the period 2010–2025, as well as on the relative stability of the forecasts for each series, rather than on a formal regression model, Granger causality model, or cointegration model. Accordingly, these claims are interpreted as dependencies consistent with the proposed system, rather than as presumed causal effects. Confirmatory causality testing (e.g., dynamic panel model or VAR/VECM with firm-level intake data) is a task for future research. Throughout the paper, the terms “determinant” and “driver” are therefore used strictly in this associational sense. The ARIMA analysis therefore demonstrates correlation and forecasting consistency only; it does not estimate the effect of AI adoption on competitiveness.

## 3 Results

### 3.1 An AI-technology framework for SME competitiveness

We first organize AI technologies relevant to small and medium-sized enterprises (SMEs) into a practical framework that links each technology class to its primary function in a small business and its qualitative cost/benefit time profile (Table 1). Of the nine technology classes currently in use—machine learning, deep learning, artificial neural networks, natural language processing (NLP), expert systems, computer vision, reinforcement learning, autonomous agents/robotics, and generative AI—three stand out as affordable, entry-level technologies with rapid ROI for resource-constrained companies: machine learning, NLP, and generative AI. Computer vision, expert systems, and autonomous agents can significantly improve efficiency, but they require extensive technical capabilities and a data infrastructure that puts them at the cutting edge. Across six operational domains—marketing and sales, customer service, financial management, logistics and supply chain, and manufacturing and human resources, the entry-level technologies are concentrated in the first three, where large amounts of data are available, integration costs are low, and return on investment is fast (in terms of lead conversion, response time, and fraud reduction). This prioritization leads to a testable proposition, which is discussed in the rest of this section: implementing accessible AI is associated with measurable improvements in the competitiveness of small and medium-sized enterprises. The remainder of the Results examines the only proposition testable with regional data (H3): whether aggregate digital adoption co-moves with small-business output.

**Table 1:** AI technology classes mapped to SME competitiveness functions and cost/time-to-value profiles.

AI technology	Principal SME function	Cost	Time-to-value	Tier
Machine learning	Demand forecasting, dynamic pricing, credit-risk screening	Low	Fast	Entry
Natural language processing	24/7 chatbots, review/sentiment analysis, translation	Low	Fast	Entry
Generative AI	Marketing content, design, copywriting	Low	Fast	Entry
Computer vision	Quality control, security, footfall analytics	Medium–High	Medium	Advanced
Expert systems	Consulting, financial/tax advice automation	Medium	Medium	Advanced
Deep learning/neural networks	Image–audio–video analysis, behavior modeling	High	Slow	Advanced
Reinforcement learning	Automated ad bidding, dynamic optimization	High	Slow	Advanced
Autonomous agents/robotics	Warehousing, packing, last-mile delivery	High	Slow	Advanced

Source: authors’ synthesis of the applied AI-for-SME literature. Tier denotes suitability for resource-constrained firms.

### 3.2 Regional small-business dynamics, 2010–2025

Based on 16 years of observations (2010–2025) of the small business sector in Uzbekistan’s Bukhara region, regional data reveal robust and rapid growth. Of particular interest are the volume of preferential bank credits (X6), which rose from UZS 51.2 billion to UZS 232.0 billion, reflecting expanding financial support for small business, and central to the digital-adoption hypothesis, the digital-technology-adoption indicator (X7), which increased from 61 to 145 per cent. Notably, X7 is not monotonic: after peaking in 2021, it declined in 2022–2023 and recovered in 2024–2025, indicating a gradual, institutionally mediated digital transformation rather than a smooth diffusion path. The persistent upward trends and structural break in 2023–2025 confirm non-stationarity and justify the use of the differenced time series approach described below.

### 3.3 Forecasts to 2030 and descriptive associations

To forecast each series for 2026–2030, ARIMA (*p*, *d*, *q*) models were used for each indicator, with orders selected by minimizing AIC (Table 2). Production volume (Y) is projected to grow from 11250.0 billion soums in 2025 to 13041.9 billion soums in 2030, an increase of approximately 16%, achieved without a sharp increase, indicating a stable, gradual growth pattern. Investment (X1), production (X2) and gross value added (X3) maintain an upward trajectory, reaching 31117.4 billion soums, 23011.6 billion soums and 40411.8 billion soums, respectively by 2030, while the number of higher-educated employees (X5) grows from 9,850 to 10,098.

**Table 2:** Bukhara small-business indicators: 2025 base values and 2026–2030 ARIMA forecasts (Y, X1–X4 and X6 in UZS bn; X5 in persons; X7 in per cent).

Year	Y	X1	X2	X3	X4	X5	X6	X7
**2025 (base)**	**11250.0**	**26200.0**	**19850.0**	**35200.0**	**5100.0**	**9,850**	**232.0**	**145.0**
2026	11587.5	27117.0	20445.5	36185.6	5253.0	9,899	237.8	146.4
2027	11935.1	28066.1	21058.9	37198.8	5410.6	9,948	243.7	147.9
2028	12293.2	29048.4	21690.7	38240.4	5572.9	9,998	249.8	149.4
2029	12662.0	30065.1	22341.4	39311.1	5740.1	10,048	256.0	150.9
2030	13041.9	31117.4	23011.6	40411.8	5912.3	10,098	262.4	152.4

Bold values indicate the 2025 base-year observed data; all other values are ARIMA forecasts for 2026–2030. Source: authors’ computation. X6, volume of preferential bank credits; X7, digital-technology-adoption indicator.

Crucially for the AI adoption hypothesis, the digital-technology-adoption indicator (X7, to 152.4%) and preferential credit volume (X6, to UZS 262.4 bn) continue to grow over the observation period. The analysis of the indicators shows that X1, X2, X3 and X6 are closely related to production, while innovation spending (X4) and digital adoption (X7) display the most persistent upward trajectories and are interpreted, descriptively, as indicators whose continued growth accompanies output growth beyond the near term. In other words, the digital adoption indicator is not a passive correlate of growth, but one of the factors related to its sustainability – an association consistent with the framework’s proposition that AI/digital adoption supports SME competitiveness.

### 3.4 Model quality

The model diagnostics are presented in Table 3. The forecasting accuracy for the human-capital indicator is the highest (X5: MAPE ≈ 3.0%, ARIMA (2, 0, 1)), while for output it is acceptable (Y: MAPE ≈ 9.2%, ARIMA (1, 0, 2)). The Ljung-Box statistic shows that the residuals are virtually free of autocorrelation for all indicators (*p* ≥ 0.97). However, several series show non-normal residuals (low Jarque-Bera *p*-values), reflecting outliers and a structural break in the 2023–2025 period; the exception is the digital adoption indicator (X7), which has residuals closest to a normal distribution (JB *p* ≈ 0.63). The investment series (X1) is the most challenging to forecast (MAPE ≈ 189%): the series collapses in 2023 and rebounds sharply in 2024, and because MAPE divides by actual values, the near-zero 2023 observation mechanically inflates the percentage error. X1 point forecasts are therefore reported for completeness only and support no substantive claim in this paper. Overall, the diagnostic results confirm the core output and employment forecasts but warrant a cautious interpretation of the more volatile indicators. They also support the paper’s central association: across all indicators, digital adoption is both linked to long-run growth and statistically the best-behaved series—consistent with, though not proof of, the proposition that low-cost AI adoption accompanies SME competitiveness in developing countries (see Figure 1).

**Table 3:** ARIMA model selection and quality metrics by indicator.

Indicator	ARIMA	AIC	BIC	MAE	RMSE	MAPE (%)	Ljung–Box *p*	JB *p*
Y	(1, 0, 2)	267.44	271.30	352.15	796.19	9.22	1.000	0.0003
X1	(1, 0, 0)	330.47	332.79	2645.00	6004.88	189.10	1.000	0.0000
X2	(2, 0, 1)	270.31	274.18	930.72	2074.98	29.91	1.000	0.0000
X3	(2, 0, 0)	300.10	303.19	1689.88	3273.89	44.44	1.000	0.0000
X4	(2, 0, 2)	243.31	247.95	256.80	423.62	27.06	0.998	0.0064
X5	(2, 0, 1)	237.43	241.29	228.14	494.93	3.02	1.000	0.0000
X6	(2, 0, 0)	86.66	89.75	8.26	27.01	14.46	1.000	0.0000
X7	(0, 0, 1)	163.06	165.38	26.26	32.63	29.22	0.971	0.6255

Source: authors’ computation. AIC/BIC, information criteria; MAE/RMSE, absolute and root-mean-square error; MAPE, mean absolute percentage error; JB, Jarque–Bera normality test.

**Figure 1:**
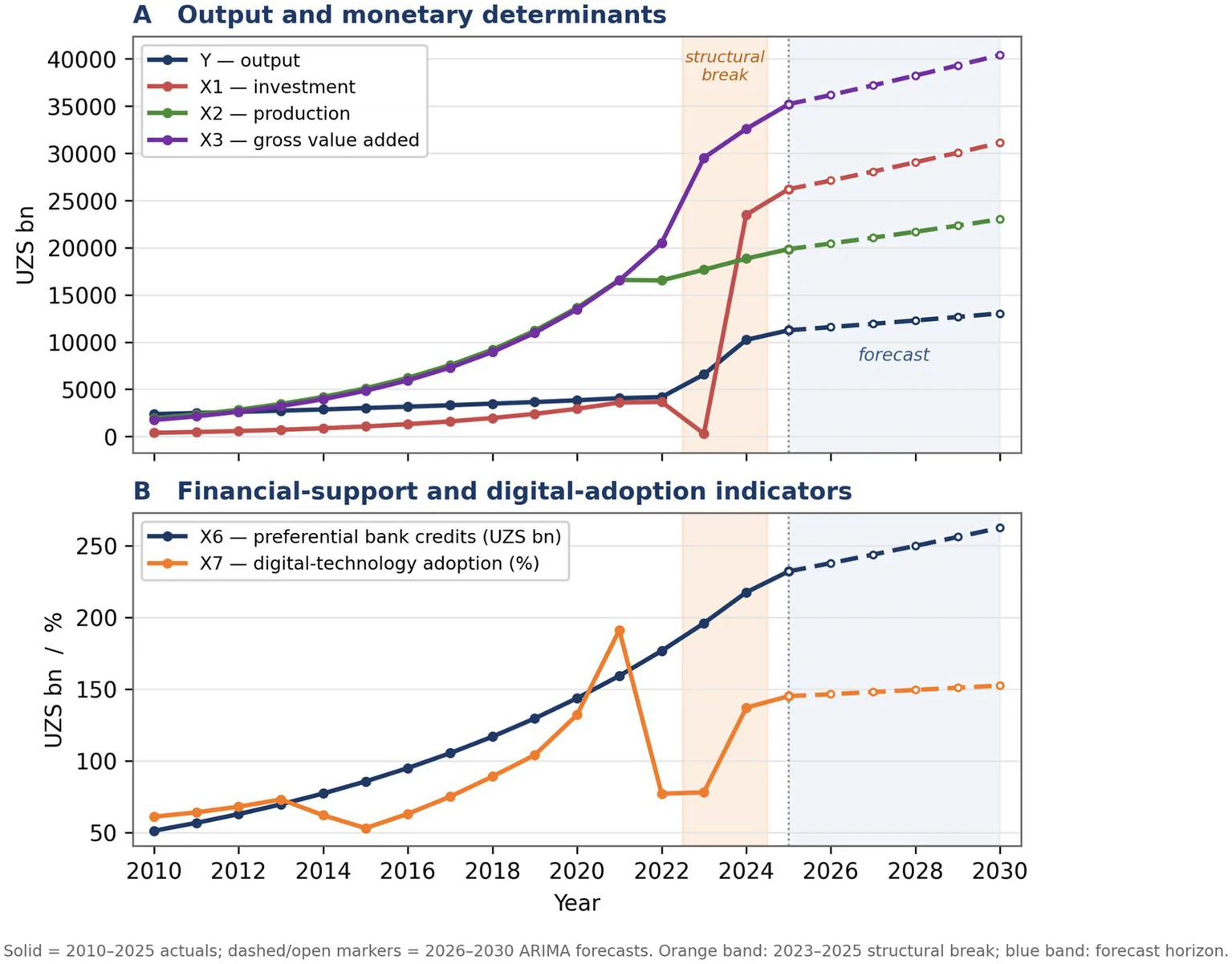
Trajectories of Bukhara small-business indicators, 2010–2030. **(A)** Output (Y) and monetary determinants—investment (X1), production (X2), and gross value added (X3), UZS bn. **(B)** Financial-support and digital-adoption indicators—preferential bank credits (X6, UZS bn) and digital-technology adoption (X7, per cent). Solid lines with filled markers are 2010–2025 actuals; dashed lines with open markers are 2026–2030 ARIMA forecasts. The orange band marks the 2023–2025 structural break and the blue band the forecast horizon. The deep V in X1 (2023) and the non-monotonic X7 path (2021 peak, 2022–2023 dip, recovery) illustrate the volatility reflected in the model diagnostics (Table 3).

## 4 Discussion

The research links the applied AI concept for SMEs with a clear regional forecast analysis, and both groups converge on a cogent proposition: for companies with limited capabilities in developing economies, accessible AI is not a secondary add-on but a measurable correlate of growth. This proposition is supported by the findings. First, machine learning, natural language processing (NLP), and generative AI are beginning to achieve a low-cost, high-return level of operational focus on marketing, customer service, and financial management channels that support SME competitiveness. Second, in the Bukhara region of Uzbekistan, small-business output is projected to grow by approximately 16% by 2030 on a stable trajectory, with digital-technology adoption (X7) and preferential credit support (X6) emerging as long-run indicators associated with this growth.

### 4.1 Theoretical implications

Classical competitiveness theory specifies where advantage comes from but not which capability delivers it: Porter’s (1980) positioning identifies cost and differentiation channels, Barney’s (2000) resource-based view explains why valuable and rare resources matter, and Teece et al.’s (1997) dynamic capabilities describe how firms reconfigure resources under change. Our framework supplies the missing operational layer by mapping each AI technology class to a competitiveness function and ranking it by cost and time-to-value. This complements, rather than replaces, theory: machine learning and NLP become the concrete sensing-and-seizing capabilities that dynamic-capabilities theory leaves abstract, echoing recent evidence that AI adoption itself functions as a dynamic capability for SME sustainability (Cimino et al., 2025). It also reframes the much-discussed “AI divide” (OECD, 2025; Yesuf and Fields, 2025): because the highest-return technologies for SMEs are also the cheapest, the divide is driven less by the cost of technology than by readiness, skills, and institutional support (Faiz et al., 2024; Badghish and Soomro, 2024; Hoang, 2024).

The empirical strand turns the framework’s central proposition from assertion into a testable association, and three features make the case strong. First, the digital-adoption indicator does not merely co-move with output; together with preferential credit support, it behaves as a long-run determinant that sustains growth beyond the immediate horizon—consistent with firm-level evidence that AI in marketing improves SME performance in emerging economies (Abrokwah-Larbi and Awuku-Larbi, 2024) and that digitalization drives innovation outcomes (Luo et al., 2023). Second, among all eight series, X7 is the most statistically well-behaved, with residuals closest to normality; the indicator carrying the AI signal is therefore also the one the model captures most reliably. Third, the convergence is independent: the qualitative framework and the quantitative forecast were constructed from different inputs, yet point in the same direction. We are deliberate about the epistemic status of this result; it is an association consistent with the framework, not an estimated causal effect (see Section 4.4).

### 4.2 Policy implications

The findings carry an unusually actionable policy edge. Because the entry-tier technologies are simultaneously the most affordable and the highest-return, raising SME digital adoption is potentially a low-cost, high-leverage lever for regional competitiveness—precisely the lever Uzbekistan’s 2024 National AI Strategy seeks to pull through its 2030 targets for AI products and services (UNDP, 2025; Yakhshiboev, 2025). The non-monotonic adoption path we observe—a 2021 peak, a 2022-2023 dip, then recovery is itself informative: it suggests diffusion is governed less by technology availability than by institutional support such as subsidies, incubators, targeted credit, and skills programs, mirroring barriers documented for emerging-economy SMEs (Shahadat et al., 2023; Shao et al., 2025). Sequencing matters: targeting the affordable entry tier first lets resource-constrained firms capture early returns and accumulate the readiness needed for advanced applications, a logic that also aligns with the augmentation-over-automation emphasis now prominent in the AI-and-work literature (Bastida et al., 2025; Garcia and Kwok, 2025; Do et al., 2025). Because the underlying evidence is associational, these recommendations are indicative rather than prescriptive: they identify where policy attention has the greatest likely leverage, not guaranteed effects.

### 4.3 Limitations

The analysis is bounded by seven limitations, stated in full so that the results are not over-read. First, the sample is very small (*n* = 16 annual observations), which limits statistical power, widens forecast uncertainty, and precludes out-of-sample validation: a holdout set would leave too few observations for estimation, so all accuracy metrics are in-sample and genuine predictive ability remains undemonstrated; rolling-origin evaluation becomes feasible only as longer series accumulate. Second, the 2023–2025 structural break and the partly estimated 2025 base-year values reduce forecast reliability precisely at the forecast origin; ARIMA is therefore trusted only over the capped five-year horizon. Third, the most volatile series—above all investment (X1, MAPE ≈ 189%) are reported for completeness and support no substantive claim. Fourth, the design permits no causal identification: the models are univariate, contain no control variables, and cannot rule out confounders—aggregate economic growth, public investment, infrastructure development, or policy reforms could plausibly drive both digital adoption (X7) and output (Y); reverse causality is equally plausible, since more competitive firms may adopt digital technologies earlier, so the observed co-movement may reflect endogenous selection rather than any effect of adoption. Fifth, the data are sector aggregates that mask firm-level heterogeneity: they cannot show whether adopting firms outperform non-adopters, and X7—a broad digital-adoption proxy without a published component-level methodology cannot isolate AI-specific uptake or distinguish the framework’s technology classes, so H1-H2 remain untested. Sixth, external validity is severely limited: the evidence comes from a single region of a single country, and any extension to other emerging economies is suggestive only. Seventh, ARIMA is a classical univariate statistical method; the contribution of this paper is the framework-to-evidence link, not methodological novelty. These constraints define, rather than diminish, the study’s role: it establishes a documented regional association and explicit hypotheses (H1–H3) for confirmatory firm-level research.

Three extensions follow directly. Pairing the framework with firm-level AI-adoption surveys would convert the proxy indicator into a measured construct and enable a causal test of the adoption—competitiveness link, using dynamic panel-data estimators (e.g., system GMM) or quasi-experimental designs. Multivariate or machine-learning forecasters—gradient-boosted trees, LSTM networks, or hybrid ARIMA-ML models could be benchmarked against the transparent ARIMA baseline used here, building on comparative evidence regarding ARIMA versus machine-learning accuracy (Kontopoulou et al., 2023; Balungu and Kumar, 2024). Finally, replication across other Uzbek regions and additional emerging economies would test the framework’s external validity and refine the entry-tier prioritization for different institutional contexts. As longer series accumulate, rolling-origin out-of-sample evaluation should complement the in-sample diagnostics reported here.

In conclusion, a small and affordable set of AI technologies offers resource-constrained SMEs a credible potential path to competitiveness, and regional data from Bukhara are consistent with the view that digital adoption is not a passive correlate but one of the factors associated with the sustainable growth of small businesses. By identifying the AI capabilities that each competitiveness channel provides and testing this logic with a transparent regional forecast, this study provides a practical, evidence-based framework for AI adoption policy in developing countries and a clear agenda for subsequent work on causal analysis at the enterprise level.

## Data Availability

The original contributions presented in the study are included in the article/supplementary material, further inquiries can be directed to the corresponding author.
